# Advances in COVID-19 Vaccines and New Coronavirus Variants

**DOI:** 10.3389/fmed.2022.888631

**Published:** 2022-07-08

**Authors:** Mengchen Liu, Yunqiao Li

**Affiliations:** Department of Geriatrics, Union Hospital, Tongji Medical College, Huazhong University of Science and Technology, Wuhan, China

**Keywords:** COVID-19, SARS-CoV-2, variant, omicron, vaccine, effectiveness

## Abstract

With the successful development of the Corona Virus Disease 2019 (COVID-19) vaccines and increased vaccination coverage, great progress in global outbreak control has been made in several countries. However, new coronavirus variants emerge and their rapid spread, causing a new wave of economic and social upheaval worldwide. The spread of new coronavirus variants poses a new and enormous challenge to vaccination and pandemic control, so further studies to explore and develop vaccines for the prevention and control virus infection are warranted. In this review, we provide an overview of the most prevalent variants including Omicron, and explore the effectiveness of COVID-19 vaccines against related variants to better understand existing vaccines and to facilitate improved research into new vaccines. In addition, this review discusses existing strategies to increase vaccine efficacy and introduces novel vaccines by the non-injection route.

## Introduction

In March 2020, the World Health Organization (WHO) announced a new coronavirus named Severe Acute Respiratory Syndrome Coronavirus 2 (SARS-CoV-2) causing a global pandemic. Since then, nearly 400 million people worldwide have been infected by it, resulting in more than 5.7 million deaths (1). Some progress has been made in controlling the epidemic with the development and production of the COVID-19 vaccines and mass vaccination. Nevertheless, an emerging SARS-CoV-2 variant was first reported in the United Kingdom in late 2020, and nowadays there are different mutations and variants prevalent around the world (2). The virus is constantly updating and mutating at an unpredictable rate, especially the newly reported Variant Omicron by the WHO (3, 4) which is gradually replacing the Delta variant and leading a new wave of outbreaks in several countries (1). The emergence of SARS-CoV-2 variants not only hindered the control of global outbreaks, but also poses new challenges to the effectiveness of existing vaccines and the development of new vaccines, so there is a need to further explore and develop vaccines for the prevention and control virus infection.

Current research on SARS-CoV-2 revolves around several aspects such as new variants and the effectiveness of the COVID-19 vaccines against the variants, but relevant trials and information are still being further studied and updated. Since certain variants have induced world-wide fears due to their ability to be highly infectious or cause severe disease, this paper details them and summarizes the effectiveness of two COVID-19 vaccines against them in order to better understand existing vaccines and to facilitate improved research on new vaccines. In addition, how to improve the effective resistance of vaccines against variants is a hot issue worldwide. In this review, we explore existing strategies to maintain or improve vaccine effectiveness and discuss the explanation of novel non-injection route COVID-19 vaccines.

## SARS-CoV-2 and Variants

The SARS-CoV-2 is a single-stranded positive-stranded Ribonucleic Acid (RNA) virus belonging to the genus β-coronavirus. SARS-CoV-2 contains 4 structural proteins and 16 non-structural proteins, of which the structural proteins include nucleoprotein, envelope, matrix protein, and spike (S) protein (2). The S protein is further divided into the amino terminal (N-terminal) S1 subunit, which is responsible for viral receptor binding, and the Carboxy terminal (C-terminal) S2 subunit, which facilitates viral-cell membrane fusion. S1 subunit includes two structures: amino acid N-terminal domain (NTD) and receptor binding domain (RBD) (5). The RBD is the top exposed loop of the S protein and is responsible for binding to the host cell receptor angiotensin-converting enzyme 2 (ACE2), which is the main target of neutralizing antibodies (6).

Usually, under natural selection, RNA viruses have a high rate of nucleotide substitution and are prone to mutations due to errors during replication, which subsequently give rise to a rapidly evolving viral population. This characteristic may lead to the accumulation of amino acid mutations, which in turn affect or alter the pathogenicity and transmissibility of the virus (7). Similarly, SARS-CoV-2 is susceptible to genetic evolution as it continues to adapt to new human hosts, leading to the emergence of multiple variants over time (8). In November 2020, the new variant Alpha (B.1.1.7) was first reported in the United Kingdom (UK). Since then, diverse new variants have been reported in various countries of the world, including South Africa, Brazil, and India, and the number is still increasing. The emergence of new coronavirus variants has changed the dynamics of the pandemic and greatly impacted public health.

There are various methods for naming and classify new variants of SARS-CoV-2, including Nextstrain typing, Pango genealogy nomenclature, and Global Initiative of Sharing All Influenza Data (GISAID) typing, among others (9). For the purpose of public understanding and information dissemination, WHO divided the variants into two categories (10): variants of concern (VOC), which include Alpha (B.1.1.7), Beta (B.1.351), Gamma (P.1), Delta (B.1.617.2), and Omicron (B.1. 1.529), and variants of interest (VOI) includes Eta, Iota, Kappa, Lambda, and Mu (Table 1). VOCs have received focused attention and detection worldwide owing to their potential to increase transmissibility and cause exacerbations.

**Table 1:** SARS-CoV-2 variants.

**Classification**	**WHO label**	**Pango lineage**	**Nextstrain clade**	**GISAID clade**	**Earliest record time**	**The first detection area**
VOC	Alpha	B.1.1.7	20I (V1)	GRY	2020-09	United Kingdom
	Beta	B.1.351	20H (V2)	GH/501Y.V2	2020-05	South Africa
		B.1.351.2				
		B.1.351.3				
	Gamma	P.1	20J (V3)	GR/501Y.V3	2020-11	Brazil
		P.1.1				
		P.1.2				
	Delta	B.1.617.2	21A, 21I, 21J	G/478K.V1	2020-10	India
		AY.1				
		AY.2				
		AY.3				
	Omicron	B.1.1.529	21K, 21L 21M	GK	2021-11	Multiple countries
		BA.1				
		BA.1.1				
		BA.2				
		BA.3				
VOI	Eta	B.1.525	21D	G/484K.V3	2020-12	Multiple countries
	Iota	B.1.526	21F	GH/253G.V1	2020-11	United States of America
	Kappa	B.1.617.1	21B	G/452R.V3	2020-10	India
	Lambda	C.37	21G	GR/452Q.V1	2020-12	Peru
	Mu	B.1.621	21H	GH	2021-01	Colombia

Alpha variant emerged in November 2020 (dating back as far as August) in southeastern England, then spread throughout the UK and eventually prevalent in over 100 countries. Compared to the original strain, it has 17 mutation sites, of which 8 mutations are located in the S protein. This kind of variants spreads much faster than other variants and may lead to exacerbation and increased mortality (11). Beta variant was first discovered in South Africa in late 2020. The results of gene sequencing show that Beta has 10 mutation sites on the S protein, of which three mutations are located in the RBD, N501Y, E484K, and K417N, respectively (12).

Gamma variant was first detected in Brazil in late 2020 and early 2021. Gamma variant contains 11 mutations in the S protein. The mutations include K417T, E484K, and N501Y in RBD, and L18F, T20N, P26S, D138Y, and R190S in NTD. It is twice as contagious as the original coronavirus strain and has the possibility to cause secondary infection (13).

In late 2020 and March 2021, the variant of B.1.617 was found in Maharashtra, India, and named by WHO as Delta. Two mutations in Delta, L452R and E484Q, have received significant attention by reason of their location in the RBD and their association with increased viral transmissibility and affinity for ACE2 (14). From March 2021, lineage B.1.617.2 dominates in India and is the main variant causing the second wave of outbreaks in India. Delta is 50% more infectious than Alpha and has extended to more than 120 countries (15).

Omicron was first identified in Botswana, Africa, and was first reported to WHO from South Africa on 24 November 2021.WHO acted quickly, and officially designated it as Omicron variant 2 days later, on 26 November 2021, and classified it as a VOC (16). As far as the published sequences of the relevant genes are known, Omicron has a large number of mutations, with more than 30 mutations in the protein alone (17). The number of mutation sites in the RBD region of the ACE2 receptor is 15, which is several times more than the most popular Delta variant (18, 19). This variant, which contains a large number of variants, is receiving global attention (20). WHO statement mentioned that this variant has an increased risk of reinfection compared to other VOCs. More worryingly, Omicron may weaken the protective efficacy of the COVID-19 vaccines, according to a report published in Nature (21).

## Evaluation of the Effectiveness of Existing Vaccines Against Variants

Due to the nature of the transmission of SARS-CoV-2 virus, close tracking and detection of mutations and variants of the virus is a very important step so as to quickly detect new changes and assess their possible impact. Numerous researchers worldwide are sequencing emerging new variants and sharing the results on public databases such as GISAID, and this international collaboration has contributed to promote in-depth research on the variants (22).

In addition to ongoing surveillance of new SARS-CoV-2 variants, there is a need to assess whether existing vaccines are losing efficacy against variants and to determine whether improved or new vaccines are necessary to restore efficacy (23). Nevertheless, assessing the effectiveness of the COVID-19 vaccine against variants is complex for the reason that the basic knowledge of SARS-CoV-2 and its variants is still being refined, and therefore the efficacy results of SARS-CoV-2 vaccines must be evaluated objectively and impartially with scientific rigor to comprehend their combined ability and clinical value (24). The currently widely accepted design approach for assessing vaccine efficacy is the test-negative case-control study design, which is also the preferred plan for evaluating influenza vaccine effectiveness. A clear advantage of this approach is that it allows control for bias because of differences in medical behavior between vaccine unvaccinated and vaccinated individuals (25). Since many approved vaccines are still in clinical trials or in small-scale use, in this paper we only use the BNT162b2 vaccine (Pfizer/BioNTech) and the ChAdOx1 nCoV-19 vaccine (AstraZeneca), which are widely used worldwide, as examples to collect and evaluate the efficacy of these two vaccines against several VOCs (Table 2).

**Table 2:** Vaccine Effectiveness with BNT162b2 or ChAdOx1 nCoV-19.

**Variant**	**Vaccination**	**Vaccination status**	**Number of participants**	**Effectiveness (95% CI)**	**Time**	**Place**
				**Percent (%)**		
Alpha	BNT162b2	≥14 days after second doe	385,853	89.5 (85.9–92.3)	February 1–March 31, 2021	Qatar
			>150,000	93.7 (91.6–95.3)	October 26, 2020–May 16, 2021	England
	ChAdOx1 nCoV-19		385,853	75.0 (70.5–78.9)	February 1–March 31, 2021	Qatar
			>150,000	74.5 (68.4–79.4)	October 26, 2020–May 16, 2021	England
Beta	BNT162b2	After one dose	385,853	16.9 (10.4–23.0)	February 1–March 31, 2021	Qatar
		≥14 days after second dose		75.0 (70.5–78.9)		
	ChAdOx1 nCoV-19	>14 days after second dose	2,026	10.4 (−76.8 to −54.8)	June 24–November 9, 2020	South Africa
						
Delta	BNT162b2	After one dose	>150,000	35.6 (22.7–46.4)	October 26, 2020–May 16, 2021	England
		≥14 days after second dose		88.0 (85.3–90.1)		
		>14 days after second dose	>200,000	79 (75–82)	April 1–June 6, 2021	Scotland
	ChAdOx1 nCoV-19	After one dose	>150,000	30.0 (24.3–35.3)	October 26, 2020–May 16, 2021	England
		≥14 days after second dose		67.0 (61.3–71.8)		
		>14 days after second dose	>200,000	60 (53–66)	April 1–June 6, 2021	Scotland

### Alpha and Beta

According to the available evidence, the COVID-19 vaccines remain clinically resistant to the Alpha variant. In the United Kingdom, a study based on all COVID-19 symptomatic infected individuals between October 26, 2020 and May 16, 2021 demonstrated that for the ChAdOx1 nCoV-19 vaccine, the effectiveness of two doses of this vaccine was 74.5% (95% CI, 68.4–79.4) in the Alpha population, while after two doses of BNT162b2 vaccine, the effectiveness was 93.7% (95% CI, 91.6–95.3) (26). Similarly, in Qatar, the effectiveness of BNT162b2 vaccine was 89.5% for any registered Alpha variant infection and 97.4% for severe disease (27).

In Beta-dominant South Africa, AstraZeneca conducted a small (2026) phase 1/2a clinical trial focusing on the efficiency of the ChAdOx1 nCoV-19 vaccine in mildly and moderately infected patients with B.1.351. The results presented efficacy of only 10.4% (95% CI, −76.8–54.8), with a maximum of no more than 60%. This may suggest that the two-dose regimen of ChAdOx1 nCoV-19 vaccine did not show protection against mild to moderate COVID-19 (28). But this survey has a small sample size and more data are required to analyze and validate it. In Qatar, researchers collected information from 265,410 BNT162b2 two-dose vaccine recipients and showed a protection effect of 75.0% against Beta infection and 97.4% against severe disease (27).

### Delta and Omicron

As far as multiple studies have been combined, the overall effectiveness of the vaccine against the variant delta remains in height, despite the high infectivity of the variant. In Scotland, after two vaccinations, the efficacy of the BNT162b2 vaccine against delta infection (confirmed by RT-PCR) was 79% compared to 60% for the ChAdOx1 nCoV-19 vaccine (29). Another survey from England further compared the effectiveness data of BNT162b2 or ChAdOx1 nCoV-19 vaccines against delta variant with alpha variant. The data showed that the efficacy of the 2 doses of BNT162b2 vaccine against alpha and delta was 93.7 and 88.0%, respectively. The 2 doses of ChAdOx1 nCoV-19 vaccine were 74.5 and 67.0% effective against the alpha and delta variant, respectively (27). Overall, the differences between the two vaccine doses against delta and alpha variants in terms of vaccine efficacy were insignificant.

Nonetheless, in the case of the developing variant Omicron, the two-dose vaccine appeared a noteworthy decrease in protection against the Omicron variant in the available experiments. The team from China found that antibody titer levels against the Omicron variant decreased more significantly between the two vaccine doses, with neutralizing antibody titers dropping to 6, ~10-fold reduction from the original strain. Moreover, after 14 days of vaccination, 80% of specimens had neutralizing antibody levels against the Omicron variant below the lower limit of detection (30). Similarly, Pfizer's BNT162b2 vaccine study showed a significant decline in human serum neutralizing antibody titers against omicron after two doses, with ~25-fold decrease compared to the original strain (31). This certainly increases the apprehension about the presented vaccines against the SARS-CoV-2 variants.

Though, even the assessment of the value of the same vaccine against the same virus varies widely due to distinctions in region, sample size, age group and even virus subtype. For instance, a trial in the United States demonstrated 100% effectiveness of the BNT162b2 vaccine in a group of adolescents aged 12–15 years, producing a greater immune response compared to younger adults (32). In another testing that also evaluated the BNT162b2 vaccine, neutralizing antibody levels were significantly lower in older vaccine recipients (33).

Across age groups, regions, populations, the type of virus, as well as the type of variant, changes over time owing to the type of vaccine. Hence, clinically determined effective vaccines need to be reassessed from time to time in different population data sets (34). Furthermore, vaccine efficacy assessment includes not only the ability to combat viral infection or transmission, but also the effectiveness of defense against severe disease and death. Consequently, with the aim of ensuring that comparisons between different vaccines are meaningful and that the most effective vaccine candidates are configured, it is critical to establish standardized methods or systems for assessing different efficacy endpoints (24).

The appraisal of validity is a particularly crucial step in the development, production and application of vaccines. Nevertheless, a multitude of additional factors need to be considered in practical applications to allow for a more comprehensive and holistic evaluations (35). The advantages and disadvantages of the two vaccines are discussed below in a series of aspects to provide more intuition and understanding when selecting a vaccine (Table 3). BNT162b2 is an RNA vaccine that requires lower temperature storage and has higher storage requirements. ChAdOx1 nCoV-19 is a non-replicating viral vector vaccine that can be stored at 2–8°C for 6 months and is suitable for use in countries and regions where storage conditions are not stringent (36).

**Table 3:** Comparison of BNT162b2 and ChAdOx1 nCoV-19.

**Vaccine**	**Vaccine type**	**Regimen**	**Condition of storage**	**Efficacy of phase 3 (after two doses) (95% CI)**		**Vaccine implications of variants**				**Serious adverse events**
				**Symptomatic diseases**	**Severe diseases**	**Alpha**	**Beta**	**Delta**	**Omicron**	
BNT162b2	RNA-based	2 doses, 0/21 days	Stable at −80°C to−60°C; 2–8°C for 1 month	91.3 (89.0 to −93.2)	95.7 (73.9 to −99.9)	89.5 (85.9–92.3) Qatar	75.0 (70.5–78.9)	88.0 (85.3–90.1)	Antibody levels reduced by 25-fold	Lymphadenopathy, Anaphylaxis, Myocarditis, Pericarditis
ChAdOx1 nCoV-19	Non-replicating viral vector	2 doses, 0/4–12 weeks	2–8°C for 6 months	66.7 (57.4–74)	100	75.0 (70.5–78.9) Qatar	10.4 (−76.8 to −54.8)	79	NA	Thromboembolic events, Thrombosis with thrombocytopenia syndrome

In terms of effectiveness in use, BNT162b2 performed better overall compared to ChAdOx1 nCoV-19, both with respect to efficacy in phase 3 clinical trials and with respect to new variants. But ChAdOx1 nCoV-19 may be working better in patients with severe disease COVID-19 (23). With regard to safety, the main serious adverse events that occurred after BNT162b2 vaccination were Lymphadenopathy, Anaphylaxis, Myocarditis and Pericarditis, with a high incidence of Lymphadenopathy at ~78.4/100 000 (37). Serious adverse events after inoculation with ChAdOx1 nCoV-19 include Thromboembolic events, Thrombosis with thrombocytopenia syndrome, Anaphylaxis, and Guillain-Barré syndrome, of which Thrombosis with thrombocytopenia syndrome is of more concern (38, 39).

## Maintenance or Improvement of Vaccine Efficacy

Although the appearance of variants may affect the effectiveness of current vaccines against SARS-CoV-2, researchers worldwide are trying their best to explore new strategies for the purpose of maintaining or improving the efficacy of the vaccine (40). Scientists have explored not only the types of vaccines and doses administered, but also new attempts to get antibodies into the body. Considering the long development cycle of new vaccines coupled with the rapid rate of virus mutation, refining on present vaccines is a time-efficient and cost-effective approach at the same time.

### Vaccine Booster

Faced with a new wave of infections caused by the SARS-CoV-2 variant Omicron and the possibility of weakened immunity over time triggered by the COVID-19 vaccine, many countries are considering whether to provide booster doses to those who have been fully vaccinated, including the United States, Germany, and Israel. Now an increasing number of countries have begun administering additional doses, such as China and Russia, and so on (41, 42).

Generally, after vaccination the body produces neutralizing antibodies and a surge in the number of associated immune cells, but over time the antibody level slowly decreases and leaves a small percentage of memory immune cells (43). Ali Ellebedy, an immunologist from the United States, believes that booster shots not only rise neutralizing antibody levels again but also expand the pool of memory B cells, promoting a faster and stronger immune response. Pfizer supported a global trial of a third booster dose of the BNT162b2 vaccine, which showed that median neutralizing antibody titers at 1 month after the third dose were beyond 5 times in the young and middle-aged group after the second dose, while in the elderly group this number exceeds 7. Additional studies monitored predominantly mild to moderate adverse effects after the booster dose, similar to those after the second dose (44). A recent team from Yale University moved forward to analyze neutralizing antibody levels after two doses of inactivated vaccine using a booster shot and found that the booster dose was able to enlarge the magnitude and breadth of neutralization on top of the already existing antibody response and may supply more effective and durable defend against the variants of Delta and Omicron (45). Results from another team in the United States similarly corroborated that the neutralizing antibody titers against the Omicron strain were 20-fold upper after the third booster dose of the mRNA-1273 vaccine than those assessed after the second dose, with a remarkable boost in antibody levels (46).

Furthermore, certain investigations have shown that booster shots not only enhance the immune response but also reduce mortality caused by SARS-CoV-2. An experiment from Israel divided 843,208 participants into a booster group (receiving a third dose of BNT162b2 after 5 months of the second dose) and a non-booster group and weighed the mortality rate in 2 groups, showing a 90% reduction in mortality in the booster group compared to the non-booster group (47).

Nonetheless, the WHO does not support the implementation of the booster vaccine and calls for its immediate discontinuation. Similarly, an article published in The Lancet on September 13, 2021, does not approve vaccine booster dosing for three reasons. First, the paper argues that most of the studies supporting this claim are preliminary and that there is an elevated potential for confounding and selective reporting among them. Second, there may be a risk of serious adverse reactions, especially with the adenoviral vector COVID-19 vaccine—with possible immune-mediated side effects (e.g., myocarditis or Guillain-Barre syndrome)—if booster doses are used too early or too widely. Third, the availability of existing vaccines is still limited, and more lives could be saved if vaccines were made available to those who are at clear risk of serious disease and have not received any vaccine (48). Even so, some research teams disagreed with the second point of appeal, and they counted and analyzed data on adverse reactions after booster vaccination on the centers for disease control and prevention (CDC) between August 12 and September 19, 2021, and showed that there were no unexpected adverse reactions and most of them were mild or moderate after receiving the booster dose of the vaccine (49). This trial has some limitations in terms of vaccine type and time span yet. In other words, despite the fact that many institutions and organizations worldwide are conducting clinical trials of booster doses and have been tracking and testing adverse reactions to booster shots, there are still huge controversies and disagreements.

In conclusion, despite the number of studies highlighting the importance and effectiveness of third dose booster vaccines, there are still a lot of gaps in this research that need to be filled. Apart from the question of whether to use booster shots, the issues of when to use them and what populations are suitable for them are all problems that need to be addressed in depth.

### Heterologous Vaccines

Booster immunization with the original vaccine is generally considered to be a fast, effective, and straightforward way to increase antibody potency, but so-called “homologous” vaccination protocols are vulnerable to unexpected delays in production, logistics, and intervals between vaccinations (42). Thus, researchers have considered mixing and matching two different vaccines (heterologous) to make vaccination protocols more flexible: this would speed up the vaccination process and reduce the impact of any supply chain disruptions (50). An examination in the UK combining ChAdOx1 nCoV-19 and BNT162b2 vaccines reported that the combination of two different vaccines produced a broader and stronger immune response compared to ChAdOx1 nCoV-19/ChAdOx1 nCoV-19 as evidenced by higher neutralizing antibody titers and T-cell levels (51). In another study in Germany using the ChAdOx1 nCoV-19/BNT162b2 vaccine regimen, researchers further explored the role of heterologous vaccination against the variant (52). The results of this trial found that the ChAdOx1 nCoV-19/BNT162b2 combination induced 11.5 times more neutralizing antibodies than a single ChAdOx1 nCoV-19 vaccination, while this figure was only 2.9 in the homologous regimen, i.e., ChAdOx1 nCoV-19/ChAdOx1 nCoV-19, and more outstandingly, the heterologous regimen produced higher levels of neutralizing antibodies against the B.1.1.7, B.1.351, and P.1 variants (53). With the exception of the above-mentioned combinations, a team of researchers from the University of Mörö in Sweden confirmed that the amalgamation of ChAdOx1 nCoV-19 and mRNA-1273 inoculation also increased the level of neutralizing antibodies (54).

Recently, the University of Oxford, UK, published an article in The Lancet on current heterologous combination regimens of SARS-CoV-2 mRNA, adenoviral vector and protein adjuvant vaccines, with programs including ChAdOx1 nCoV-19/NVX-CoV2373, ChAdOx1 nCoV-19/mRNA-1273, ChAdOx1 nCoV-19/BNT162b2, BNT162b2/mRNA-1273, and BNT162b2/NVX-CoV2373. Consistent with previous findings, the article confirmed that the mixture vaccines were strongly resistant to the virus, especially the regimens containing mRNA vaccines (55).

Nonetheless, the time to reach effective antibody titers and the level of neutralizing antibodies produced in humans after vaccination differ by reason of the type of vaccine. The subject of how to achieve a mixture of different vaccines while ensuring that effective or even maximum antibody levels are achieved after vaccination requires further research and calculation of immune antibody levels and vaccination intervals. Moreover, because of the complexity of the immune system and the limited knowledge available to humans, the issue of whether mixing different vaccines causes potential adverse reactions or overwhelming immune responses leading to immune system-related diseases or even more serious consequences needs to be further investigated over a longer period of time and with more rigorous trials and follow-ups.

On the whole, immune efficacy is improved by increasing the dose of vaccine, whether through booster shots or a combination of vaccines. Perhaps trials of low-dose vaccines can shed new light. An experiment from the United States estimate the immune efficacy produced by the two different doses through measuring the levels of acute and memory SARS-CoV-2-specific antibodies, CD4+ T cells and CD8+ T cells in blood samples from subjects receiving either a low dose (25 μg) or a standard dose (100 μg) of mRNA-1273 vaccine (56).The findings suggest that low doses of mRNA1273 vaccine can induce the production of persistent and functional T cells and immune memory comparable to natural infection. At the same time, a number of studies have also shown that low doses of RNA vaccines have fewer immune side effects (43, 49). This research is very attractive in the context of global vaccine scarcity and multi-dose regimens.

Low-dose RNA vaccines have great prospection for future demand and application. If it can be applied in clinical practice in the future, it will not only save vaccine doses and alleviate the vaccine shortage, but also minimize the side effects associated with the vaccine. On the other hand, in practical applications, dissimilarity in different age or risk groups need to be considered, which requires advanced studies on immune efficacy and immune memory at different doses.

## Development of Novel Vaccines

Vaccination currently remains the safeguard and cornerstone of the fight against the SARS-CoV-2 variants and global health care. Although SARS-CoV-2 vaccines are being developed at an unprecedented rate, most vaccines that have been approved or are currently in the clinical trial process are almost always administered by the injectable route and are capable of inducing a systemic immune response in vaccinated individuals (57). This requires not only the operation of professionally trained medical personnel, but also the support of strictly regulated cold chain facilities. In order to break the limitations of traditional vaccines, scientists have tried and used new scientific techniques to develop novel vaccines, starting from non-specific immunity. Nowadays, the intranasal vaccines and patch vaccines have received a lot of attention.

### Intranasal Vaccines for SARS-CoV-2

Previous studies have shown that SARS-CoV-2 is a respiratory virus that is transmitted primarily by the “respiratory droplet” route, and therefore virus invasion and replication begin in the respiratory mucosa. Although the available humoral and cellular immune responses induced by SARS-CoV-2 vaccination are highly effective in humans (70–95%), there is little protection against infection of the virus in the upper respiratory tract, suggesting that vaccine recipients are still at risk of virus infection and transmission (58). Thus, some researchers designed an inhalable nanovaccine that induces mucosal immunity by mimicking the structure and pathway of viral infection. It was found that this nanovaccine induced the production of secretory immunoglobulin A (sIgA) titers that were hundreds of times higher than those of conventional injectable vaccines and provided longer protection (lasting at least 5 months) against viral entry through the upper and lower respiratory tracts (59). Several scientists have then suggested that intramuscular vaccination followed by a booster inhalation vaccine may produce a more comprehensive immune response, including prevention or reduction of viral replication in the upper and lower respiratory tracts. Intranasal vaccines are receiving increasing attention and focus from scientists worldwide. Currently, intranasal vaccines are under development or clinical trials in many countries, such as the ChAdOx1 nCoV-19 vaccine (intranasal route) in the United Kingdom, the ChAd-SARS-CoV-2-S vaccine in the United States, and the Ad5-nCoV and ZF2001 vaccines in China, among others (60–62).

Along with the strong mucosal protection induced by intranasal vaccines, there are other potential advantages. While intramuscular vaccines usually entail specialized and trained medical personnel to administer and manage, intranasal vaccines are non-invasive and self-administered, which not only saves labor for medical personnel, but makes them more acceptable to the vaccine recipient. Additionally, the vaccine can be stored at room temperature and the vaccine device is reusable, eliminating the need for refrigeration as is required for intranasal vaccines, which foster simplifying transport and storage (63).

Even if nasal inhalation vaccines hold promise for market adoption and even as an alternative to injectable vaccines, there are some hidden problems that need to be addressed. For instance, despite the fact that the mucosal immune response may play an important role in preventing viral invasion of the upper and lower respiratory tract, it may be counterproductive if it elicits an overly robust immune response, worsening lung conditions and causing enhancement of antibody-associated respiratory disease. Previous studies have demonstrated that severe COVID-19 cases are associated with an excessive immune system response caused by higher IgA titers. Therefore, careful testing and evaluation of possible IgA-induced adverse events is necessary before nasal inhalation vaccines are introduced to the marketplace (64). Also, most intranasal vaccines against SARS-CoV-2 are in early clinical trials, and pregnant women and children are not included in clinical trials for this vaccine study, so data on the safety and efficacy of this vaccine need to be investigated statistically (65).

### Patch Vaccine

The WHO announced on October 26, 2021, that they are launching the takeoff of the Solidarity Trial Vaccines (STV) project in collaboration with the Ministries of Health of Colombia, Mali, and the Philippines. The aim of the STV project is to help discover a new generation of vaccines with possibility for elaboration against SARS-CoV-2 variants, longer immune protection, non-injection routes, etc., which can also be called second-generation vaccines. The SARS-CoV-2 vaccine patch undoubtedly belongs to the second generation of vaccines (66). This vaccine was developed by a team from the University of Queensland, Australia, using a technology called the high-density microarray patch (HD-MAP), which can be administered by simply attaching the patch to the upper arm. Even though it is only 7 x 7 mm, it is distributed with 5,000 needle-like protrusions that are able to pierce the skin and deposit the vaccine into the immune-rich epidermal and dermal tissue layers without pain or skin bleeding. Academics have now completed preliminary tests in rats and have found that this approach induces a neutralizing antibody IgG and cellular immune response that is superior to the cellular and humoral immunity produced by vaccine injection. As well as inducing a higher immune response, the patch can be stored stably at room temperature for up to 1 month (67). This simple and convenient method of vaccination may also lower the threshold for vaccination, benefiting those who fear pain and needles.

Though there is a lot of scope for future development and application of the patch vaccine, it is too early to actually market it and make it available to the general public. Examination on patch vaccines developed specifically for the new coronavirus is still in the animal testing phase and has not yet entered human testing. It is reported that the University of Queensland team will initiate a Phase I clinical trial in 2022 in collaboration with a patch technology company. On the other hand, more validation is needed to determine whether the SARS-CoV-2 vaccine patch can replace the injectable vaccine or be used as an adjunct to it.

## Discussion

The transmissibility of SARS-CoV-2 and their ability to rapidly evolve and mutate have undoubtedly caused concern worldwide. In this situation, it becomes increasingly imperative to assess whether current therapies still maintain efficacy against the variants. However, recent experiments have found a significant decrease in the protection of the two-dose vaccine against the new variant of Omicron strain. Reassuringly, a number of investigations have analyzed that a third homologous or heterologous booster dose boosts the neutralizing antibody titer of the vaccine against the Omicron strain (45, 46). In attempt to preserve or boost their efficacy against new variants, institutions and organizations around the world are constantly developing new vaccines beyond the effective dose, including innovations in types and delivery methods. For instance, the World Health Organization has approved the addition of the adjuvant subunit protein vaccine NVX-CoV2373 to the emergency use list on December 20, 2021 (68).

It requires a certain amount of time for a new vaccine to be developed, introduced into the market and accepted by the general public. Vaccine costs, costs, access, frequency, duration and efficacy are all part of the thinking, especially in low- and middle-income countries (69). For example, the storage and logistics of many vaccines require ultra-cold chain conditions of −60°C to −90°C, which is a significant financial burden for low- and middle-income countries with limited resources (70). But the solutions always outweigh the difficulties.

Firstly, government departments should play a leading role by actively and innovatively disseminating knowledge about the new coronavirus and vaccines and promoting the benefits of vaccination to increase vaccination coverage. Second, ensure fair, open and transparent distribution of vaccine resources to prevent corruption in the production and distribution of vaccines in the supply chain (71). Third, it is a cost-effective way to shift to vaccines that can be stored at +2°C to +8°C, reduce the cost of vaccination and promote vaccination motivation. Fourth, new nasal aspirate and patch vaccines have been developed to address the route and frequency of vaccination, both of which are highly acceptable and easy to use (54, 60). Fifth, a multi-strategy system involving social, political, and cultural aspects for individuals is necessary to address the challenges of vaccine hesitancy (72). For example, promoting public participation in clinical trials or promoting the involvement of community and religious leaders in vaccination campaigns (73).

Notwithstanding, the latest variant- Omicron, for example, has emerged and spread at an unexpectedly rapid rate, and new or stronger variants are likely to emerge in the future (74). What is more, the development of improved or new vaccines requires a race against time and challenges, and vaccines are not suitable for everyone, such as some people with poor innate immunity, whose protective power as well as persistence is difficult to estimate. Consequently, a single or temporary vaccination will not directly stop the spread of the variant and the epidemic, and stopping a COVID-19 pandemic requires a multi-path approach and strategy to fight the virus—vaccines, preventive and control measures, and therapeutic drugs are all needed. Although prevention and control measures may sound like a lofty and difficult concept to implement, they are manifested in all aspects of life, for example, washing hands regularly, wearing masks, and maintaining social distance. On the other hand, prevention and control measures cannot remain at the level of advocacy, but must be implemented at the grassroots level and at the individual level in every country, and must be prepared for long-term implementation.

There has been significant recent research advancement in the area of therapeutic agents, with the world's first approved oral antiviral for new coronavirus, Molnupiravir, approved in the United Kingdom on November 4, 2021, which significantly reduced the risk of hospitalization or death in patients with neoconiosis by ~50% according to interim phase III clinical data (75). A recent study further found that early use of Molnupiravir reduced the risk of hospitalization or death in non-hospitalized, unvaccinated adult COVID-19 patients (76). Molnupiravir is currently used to treat patients with mild to moderate COVID-19. Another antiviral drug, paxlovid, developed by Pfizer, is reported to be 89% effective in patients at risk of severe disease (77). The successful development of antiviral drugs has a great positive effect on controlling the epidemic.

In spite of antiviral drugs, there is no substitute for the role of vaccines, both in preventing infection and in reducing the rate of severe disease and death. Furthermore, although scientists have been working on the structure and pathogenesis of SARS-CoV-2, the understanding is still limited. So, continuous monitoring and consistent research is needed to enhance understanding so that prevention and treatment strategies can be improved and enhanced.

## Author Contributions

ML reviewed the literature and wrote the manuscript. YL participated in writing the manuscript and revising the final manuscript. Both authors contributed to the article and approved the submitted version.

## Conflict of Interest

The authors declare that the research was conducted in the absence of any commercial or financial relationships that could be construed as a potential conflict of interest.

## Publisher's Note

All claims expressed in this article are solely those of the authors and do not necessarily represent those of their affiliated organizations, or those of the publisher, the editors and the reviewers. Any product that may be evaluated in this article, or claim that may be made by its manufacturer, is not guaranteed or endorsed by the publisher.

## References

[1] Our World in Data. Coronavirus (COVID-19) Cases. (2022). Available online at: https://ourworldindata.org/covid-cases (accessed August 12, 2022).

[2] Tao K Tzou PL Nouhin J Gupta RK de Oliveira T Kosakovsky PS. The biological and clinical significance of emerging SARS-CoV-2 variants. Nat Rev Genet. (2021) 22:757–73. 10.1038/s41576-021-00408-x 34535792 PMC8447121

[3] Karim S Karim QA. Omicron SARS-CoV-2 variant: a new chapter in the COVID-19 pandemic. Lancet. (2021) 398:2126–8. 10.1016/S0140-6736(21)02758-6 34871545 PMC8640673

[4] Singhal T. The emergence of omicron: challenging times are here again!. Indian J Pediatr. (2022) 89:490–6. 10.1007/s12098-022-04077-4 35025038 PMC8756165

[5] Walls AC Park YJ Tortorici MA Wall A McGuire AT Veesler D. Structure, function, and antigenicity of the SARS-CoV-2 spike glycoprotein. Cell. (2020) 181:281–92. 10.1016/j.cell.2020.02.058 32155444 PMC7102599

[6] Ke Z Oton J Qu K Cortese M Zila V McKeane L. Structures and distributions of SARS-CoV-2 spike proteins on intact virions. Nature. (2020) 588:498–502. 10.1038/s41586-020-2665-2 32805734 PMC7116492

[7] Kustin T Stern A. Biased mutation and selection in RNA viruses. Mol Biol Evol. (2021) 38:575–88. 10.1093/molbev/msaa247 32986832 PMC7543401

[8] Callaway E. The coronavirus is mutating - does it matter? Nature. (2020) 585:174–7. 10.1038/d41586-020-02544-6 32901123

[9] Rambaut A Holmes EC O'Toole A Hill V McCrone JT Ruis C. A dynamic nomenclature proposal for SARS-CoV-2 lineages to assist genomic epidemiology. Nat Microbiol. (2020) 5:1403–7. 10.1038/s41564-020-0770-5 32669681 PMC7610519

[10] Aleem A Akbar Samad AB Slenker AK. Emerging variants of SARS-CoV-2 and novel therapeutics against coronavirus (COVID-19). In: StatPearls. Treasure Island (FL): StatPearls Publishing (2022). 34033342

[11] Davies NG Abbott S Barnard RC Jarvis CI Kucharski AJ Munday JD. Estimated transmissibility and impact of SARS-CoV-2 lineage B.1.1.7 in England. Science. (2021) 372:1–9. 10.1126/science.abg3055 33658326 PMC8128288

[12] Yuan M Huang D Lee CD Wu NC Jackson AM Zhu X. Structural and functional ramifications of antigenic drift in recent SARS-CoV-2 variants. Science. (2021) 373:818–23. 10.1126/science.abh1139 34016740 PMC8284396

[13] Naveca FG Nascimento V de Souza VC Corado AL Nascimento F Silva G. COVID-19 in Amazonas, Brazil, was driven by the persistence of endemic lineages and P.1 emergence. Nat Med. (2021) 27:1230–8. 10.1038/s41591-021-01378-7 34035535

[14] Cherian S Potdar V Jadhav S Yadav P Gupta N Das M. SARS-CoV-2 spike mutations, L452R, T478K, E484Q and P681R, in the second wave of COVID-19 in maharashtra, india. Microorganisms. (2021) 9:1542. 10.3390/microorganisms9071542 34361977 PMC8307577

[15] Liu Y Rocklov J. The reproductive number of the Delta variant of SARS-CoV-2 is far higher compared to the ancestral SARS-CoV-2 virus. J Travel Med. (2021) 28:1–3. 10.1093/jtm/taab124 34369565 PMC8436367

[16] World Health Organization. Classification of Omicron (B.1.1.529): SARS-CoV-2 Variant of Concern. (2021). Available online at: https://www.who.int/news/item/26-11-2021-classification-of-omicron-(b.1.1.529)-sars-cov-2-variant-of-concern (accessed November 26, 2021).

[17] Kandeel M Mohamed M Abd EH Venugopala KN El-Beltagi HS. Omicron variant genome evolution and phylogenetics. J Med Virol. (2022) 94:1627–32. 10.1002/jmv.27515 34888894 PMC9015349

[18] Flemming A. Omicron, the great escape artist. Nat Rev Immunol. (2022) 22:75. 10.1038/s41577-022-00676-6 35017722 PMC8749340

[19] Kumar S Thambiraja TS Karuppanan K Subramaniam G. Omicron and delta variant of SARS-CoV-2: a comparative computational study of spike protein. J Med Virol. (2022) 94:1641–9. 10.1002/jmv.27526 34914115

[20] Aouissi HA. Algeria's preparedness for Omicron variant and for the fourth wave of COVID-19. Glob Health Med. (2021) 3:413–4. 10.35772/ghm.2021.01117 35036625 PMC8692091

[21] Callaway E. Omicron likely to weaken COVID vaccine protection. Nature. (2021) 600:367–8. 10.1038/d41586-021-03672-3 34880488

[22] Cevik M Grubaugh ND Iwasaki A Openshaw P. COVID-19 vaccines: keeping pace with SARS-CoV-2 variants. Cell. (2021) 184:5077–81. 10.1016/j.cell.2021.09.010 34534444 PMC8445744

[23] Krause PR Fleming TR Longini IM Peto R Briand S Heymann DL. SARS-CoV-2 variants and vaccines. N Engl J Med. (2021) 385:179–86. 10.1056/NEJMsr2105280 34161052 PMC8262623

[24] Hodgson SH Mansatta K Mallett G Harris V Emary K Pollard AJ. What defines an efficacious COVID-19 vaccine? A review of the challenges assessing the clinical efficacy of vaccines against SARS-CoV-2. Lancet Infect Dis. (2021) 21:e26–35. 10.1016/S1473-3099(20)30773-8 33125914 PMC7837315

[25] Jackson ML Nelson JC. The test-negative design for estimating influenza vaccine effectiveness. Vaccine. (2013) 31:2165–8. 10.1016/j.vaccine.2013.02.053 23499601

[26] Lopez BJ Andrews N Gower C Gallagher E Simmons R Thelwall S. Effectiveness of COVID-19 vaccines against the B.1.617.2 (Delta) variant. N Engl J Med. (2021) 385:585–94. 10.1056/NEJMoa2108891 34289274 PMC8314739

[27] Abu-Raddad LJ Chemaitelly H Butt AA. Effectiveness of the BNT162b2COVID-19 vaccine against the B.1.1.7 and B.1.351 variants. N Engl J Med. (2021) 385:187–9. 10.1056/NEJMc2104974 33951357 PMC8117967

[28] Madhi SA Baillie V Cutland CL Voysey M Koen AL Fairlie L. Efficacy of the ChAdOx1 nCoV-19 COVID-19 vaccine against the B.1.351 variant. N Engl J Med. (2021) 384:1885–98. 10.1056/NEJMoa2102214 33725432 PMC7993410

[29] Sheikh A McMenamin J Taylor B Robertson C. SARS-CoV-2 Delta VOC in Scotland: demographics, risk of hospital admission, and vaccine effectiveness. Lancet. (2021) 397:2461–2. 10.1016/S0140-6736(21)01358-1 34139198 PMC8201647

[30] Ai J Zhang H Zhang Y Lin K Zhang Y Wu J. Omicron variant showed lower neutralizing sensitivity than other SARS-CoV-2 variants to immune sera elicited by vaccines after boost. Emerg Microbes Infect. (2022) 11:337–43. 10.1080/22221751.2021.2022440 34935594 PMC8788341

[31] Garcia-Beltran WF St DK Hoelzemer A Lam EC Nitido AD Sheehan ML. MRNA-based COVID-19 vaccine boosters induce neutralizing immunity against SARS-CoV-2 Omicron variant. Cell. (2022) 185:457–66. 10.1016/j.cell.2021.12.033 34995482 PMC8733787

[32] Frenck RJ Klein NP Kitchin N Gurtman A Absalon J Lockhart S. Safety, immunogenicity, and efficacy of the BNT162b2 COVID-19 vaccine in adolescents. N Engl J Med. (2021) 385:239–50. 10.1056/NEJMoa2107456 34043894 PMC8174030

[33] Muller L Andree M Moskorz W Drexler I Walotka L Grothmann R. Age-dependent immune response to the Biontech/Pfizer BNT162b2 coronavirus disease 2019 vaccination. Clin Infect Dis. (2021) 73:2065–72. 10.1093/cid/ciab381 33906236 PMC8135422

[34] Cai C Peng Y Shen E Huang Q Chen Y Liu P. A comprehensive analysis of the efficacy and safety of COVID-19 vaccines. Mol Ther. (2021) 29:2794–805. 10.1016/j.ymthe.2021.08.001 34365034 PMC8342868

[35] Fiolet T Kherabi Y MacDonald CJ Ghosn J Peiffer-Smadja N. Comparing COVID-19 vaccines for their characteristics, efficacy and effectiveness against SARS-CoV-2 and variants of concern: a narrative review. Clin Microbiol Infect. (2022) 28:202–21. 10.1016/j.cmi.2021.10.005 34715347 PMC8548286

[36] Knoll MD Wonodi C. Oxford-AstraZeneca COVID-19 vaccine efficacy. Lancet. (2021) 397:72–4. 10.1016/S0140-6736(20)32623-4 33306990 PMC7832220

[37] Barda N Dagan N Ben-Shlomo Y Kepten E Waxman J Ohana R. Safety of the BNT162b2 mRNA COVID-19 vaccine in a nationwide setting. N Engl J Med. (2021) 385:1078–90. 10.1056/NEJMoa2110475 34432976 PMC8427535

[38] Schultz NH Sorvoll IH Michelsen AE Munthe LA Lund-Johansen F Ahlen MT. Thrombosis and Thrombocytopenia after ChAdOx1 nCoV-19 vaccination. N Engl J Med. (2021) 384:2124–30. 10.1056/NEJMoa2104882 33835768 PMC8112568

[39] Lounis M Rais MA Bencherit D Aouissi HA Oudjedi A Klugarova J. Side effects of COVID-19 inactivated virus vs. adenoviral vector vaccines: experience of algerian healthcare workers. Front Public Health. (2022) 10:896343. 10.3389/fpubh.2022.896343 35651866 PMC9149155

[40] Alameh MG Tombacz I Bettini E Lederer K Sittplangkoon C Wilmore JR. Lipid nanoparticles enhance the efficacy of mRNA and protein subunit vaccines by inducing robust T follicular helper cell and humoral responses. Immunity. (2021) 54:2877–92. 10.1016/j.immuni.2021.11.001 34852217 PMC8566475

[41] Callaway E. COVID vaccine boosters: the most important questions. Nature. (2021) 596:178–80. 10.1038/d41586-021-02158-6 34354274

[42] Mallapaty S. China's COVID vaccines have been crucial - now immunity is waning. Nature. (2021) 598:398–9. 10.1038/d41586-021-02796-w 34650240

[43] Shrotri M Navaratnam A Nguyen V Byrne T Geismar C Fragaszy E. Spike-antibody waning after second dose of BNT162b2 or ChAdOx1. Lancet. (2021) 398:385–7. 10.1016/S0140-6736(21)01642-1 34274038 PMC8285117

[44] Falsey AR Frenck RJ Walsh EE Kitchin N Absalon J Gurtman A. SARS-CoV-2 neutralization with BNT162b2 vaccine dose 3. N Engl J Med. (2021) 385:1627–9. 10.1056/NEJMc2113468 34525276 PMC8461567

[45] Perez-Then E Lucas C Monteiro VS Miric M Brache V Cochon L. Neutralizing antibodies against the SARS-CoV-2 Delta and Omicron variants following heterologous CoronaVac plus BNT162b2 booster vaccination. Nat Med. (2022) 28:481–5. 10.1038/s41591-022-01705-6 35051990 PMC8938264

[46] Pajon R Doria-Rose NA Shen X Schmidt SD O'Dell S McDanal C. SARS-CoV-2 omicron variant neutralization after mRNA-1273 booster vaccination. N Engl J Med. (2022) 386:1088–91. 10.1056/NEJMc2119912 35081298 PMC8809504

[47] Arbel R Hammerman A Sergienko R Friger M Peretz A Netzer D. BNT162b2 vaccine booster and mortality due to COVID-19. N Engl J Med. (2021) 385:2413–20. 10.1056/NEJMoa2115624 34879190 PMC8728797

[48] Krause PR Fleming TR Peto R Longini IM Figueroa JP Sterne J. Considerations in boosting COVID-19 vaccine immune responses. Lancet. (2021) 398:1377–80. 10.1016/S0140-6736(21)02046-8 34534516 PMC8437678

[49] Hause AM Baggs J Marquez P Myers TR Gee J Su JR. COVID-19 vaccine safety in children aged 5-11 years - United States, November 3-December 19, 2021. MMWR Morb Mortal Wkly Rep. (2021) 70:1755–60. 10.15585/mmwr.mm705152a1 34968370 PMC8736274

[50] Ledford H. Could mixing COVID vaccines boost immune response? Nature. (2021) 590:375–6. 10.1038/d41586-021-00315-5 33547431

[51] Liu X Shaw RH Stuart A Greenland M Aley PK Andrews NJ. Safety and immunogenicity of heterologous versus homologous prime-boost schedules with an adenoviral vectored and mRNA COVID-19 vaccine (Com-COV): a single-blind, randomised, non-inferiority trial. Lancet. (2021) 398:856–69. 10.1016/S0140-6736(21)01694-9 34370971 PMC8346248

[52] Barros-Martins J Hammerschmidt SI Cossmann A Odak I Stankov MV Morillas RG. Immune responses against SARS-CoV-2 variants after heterologous and homologous ChAdOx1 nCoV-19/BNT162b2 vaccination. Nat Med. (2021) 27:1525–9. 10.1038/s41591-021-01449-9 34262158 PMC8440184

[53] Schmidt T Klemis V Schub D Mihm J Hielscher F Marx S. Immunogenicity and reactogenicity of heterologous ChAdOx1 nCoV-19/mRNA vaccination. Nat Med. (2021) 27:1530–5. 10.1038/s41591-021-01464-w 34312554 PMC8440177

[54] Normark J Vikstrom L Gwon YD Persson IL Edin A Bjorsell T. Heterologous ChAdOx1 nCoV-19 and mRNA-1273 vaccination. N Engl J Med. (2021) 385:1049–51. 10.1056/NEJMc2110716 34260850 PMC8314734

[55] Stuart A Shaw RH Liu X Greenland M Aley PK Andrews NJ. Immunogenicity, safety, and reactogenicity of heterologous COVID-19 primary vaccination incorporating mRNA, viral-vector, and protein-adjuvant vaccines in the UK (Com-COV2): a single-blind, randomised, phase 2, non-inferiority trial. Lancet. (2022) 399:36–49. 10.1016/S0140-6736(21)02718-5 34883053 PMC8648333

[56] Mateus J Dan JM Zhang Z Rydyznski MC Lammers M Goodwin B. Low-dose mRNA-1273 COVID-19 vaccine generates durable memory enhanced by cross-reactive T cells. Science. (2021) 374:j9853. 10.1126/science.abj9853 34519540 PMC8542617

[57] Rubin R. Trying to block SARS-CoV-2 transmission with intranasal vaccines. JAMA. (2021) 326:1661–3. 10.1001/jama.2021.18143 34647956

[58] Rubin R. COVID-19 vaccine nasal spray. JAMA. (2021) 326:1138. 10.1001/jama.2021.14996 34581751

[59] Zheng B Peng W Guo M Huang M Gu Y Wang T. Inhalable nanovaccine with biomimetic coronavirus structure to trigger mucosal immunity of respiratory tract against COVID-19. Chem Eng J. (2021) 418:129392. 10.1016/j.cej.2021.129392 33762883 PMC7972832

[60] van Doremalen N Purushotham JN Schulz JE Holbrook MG Bushmaker T Carmody A. Intranasal ChAdOx1 nCoV-19/AZD1222 vaccination reduces viral shedding after SARS-CoV-2 D614G challenge in preclinical models. Sci Transl Med. (2021) 13:1–15. 10.1126/scitranslmed.abh0755 34315826 PMC9267380

[61] Hassan AO Shrihari S Gorman MJ Ying B Yaun D Raju S. An intranasal vaccine durably protects against SARS-CoV-2 variants in mice. Cell Rep. (2021) 36:109452. 10.1016/j.celrep.2021.109452 34289385 PMC8270739

[62] Wu S Huang J Zhang Z Wu J Zhang J Hu H. Safety, tolerability, and immunogenicity of an aerosolised adenovirus type-5 vector-based COVID-19 vaccine (Ad5-nCoV) in adults: Preliminary report of an open-label and randomised phase 1 clinical trial. Lancet Infect Dis. (2021) 21:1654–64. 10.1016/S1473-3099(21)00396-0 34324836 PMC8313090

[63] Heida R Hinrichs WL Frijlink HW. Inhaled vaccine delivery in the combat against respiratory viruses: a 2021 overview of recent developments and implications for COVID-19. Expert Rev Vaccines. (2021) 20:1–18. 10.1080/14760584.2021.1903878 33749491

[64] Yu HQ Sun BQ Fang ZF Zhao JC Liu XY Li YM. Distinct features of SARS-CoV-2-specific IgA response in COVID-19 patients. Eur Respir J. (2020) 56:2001526. 10.1183/13993003.01526-2020 32398307 PMC7236821

[65] Chavda VP Vora LK Pandya AK Patravale VB. Intranasal vaccines for SARS-CoV-2: from challenges to potential in COVID-19 management. Drug Discov Today. (2021) 26:2619–36. 10.1016/j.drudis.2021.07.021 34332100 PMC8319039

[66] Korkmaz E Balmert SC Sumpter TL Carey CD Erdos G Falo LJ. Microarray patches enable the development of skin-targeted vaccines against COVID-19. Adv Drug Deliv Rev. (2021) 171:164–86. 10.1016/j.addr.2021.01.022 33539853 PMC8060128

[67] McMillan C Choo J Idris A Supramaniam A Modhiran N Amarilla AA. Complete protection by a single-dose skin patch-delivered SARS-CoV-2 spike vaccine. Sci Adv. (2021) 7:j8065. 10.1126/sciadv.abj8065 34714668 PMC8555896

[68] Dunkle LM Kotloff KL Gay CL Anez G Adelglass JM Barrat HA. Efficacy and safety of NVX-CoV2373 in adults in the united states and mexico. N Engl J Med. (2022) 386:531–43. 10.1056/NEJMoa2116185 34910859 PMC8693692

[69] Chootipongchaivat S Chantarastapornchit V Kulpeng W Ceria JA Tolentino NI Teerawattananon Y. Vaccination program in a resource-limited setting: a case study in the Philippines. Vaccine. (2016) 34:4814–9. 10.1016/j.vaccine.2016.08.014 27531410 PMC5022401

[70] Gashaw T Hagos B Sisay M. Expected impacts of COVID-19: considering resource-limited countries and vulnerable population. Front Public Health. (2021) 9:614789. 10.3389/fpubh.2021.614789 34026704 PMC8131657

[71] Persad G Peek ME Emanuel EJ. Fairly prioritizing groups for access to COVID-19 vaccines. JAMA. (2020) 324:1601–2. 10.1001/jama.2020.18513 32910182

[72] Lawal L Aminu BM Murwira T Avoka C Yusuf MS Harrison OI. Low coverage of COVID-19 vaccines in Africa: current evidence and the way forward. Hum Vaccin Immunother. (2022) 18:2034457. 10.1080/21645515.2022.2034457 35240908 PMC9009957

[73] Finney RL Zhu X Leppin AL Ridgeway JL Swift MD Griffin JM. Evidence-Based strategies for clinical organizations to address COVID-19 vaccine hesitancy. Mayo Clin Proc. (2021) 96:699–707. 10.1016/j.mayocp.2020.12.024 33673921 PMC7772995

[74] Graham F. Daily briefing: omicron is unlikely to end the pandemic. Nature. (2022). 10.1038/d41586-022-00325-x. [Epub ahead of print]. 35121812

[75] Mahase E. COVID-19: molnupiravir reduces risk of hospital admission or death by 50% in patients at risk, MSD reports. BMJ. (2021) 375:n2422. 10.1136/bmj.n2422 34607801

[76] Jayk BA Gomes DSM Musungaie DB Kovalchuk E Gonzalez A Delos RV. Molnupiravir for oral treatment of COVID-19 in nonhospitalized patients. N Engl J Med. (2022) 386:509–20. 10.1056/NEJMoa2116044 34914868 PMC8693688

[77] Mahase E. COVID-19: Pfizer's paxlovid is 89% effective in patients at risk of serious illness, company reports. BMJ. (2021) 375:n2713. 10.1136/bmj.n2713 34750163

